# How to Obtain the Maximum Properties Flexibility of 3D Printed Ketoprofen Tablets Using Only One Drug-Loaded Filament?

**DOI:** 10.3390/molecules26113106

**Published:** 2021-05-22

**Authors:** Jolanta Pyteraf, Witold Jamróz, Mateusz Kurek, Joanna Szafraniec-Szczęsny, Daniel Kramarczyk, Karolina Jurkiewicz, Justyna Knapik-Kowalczuk, Jacek Tarasiuk, Sebastian Wroński, Marian Paluch, Renata Jachowicz

**Affiliations:** 1 Department of Pharmaceutical Technology and Biopharmaceutics, Jagiellonian University Medical College, Medyczna 9, 30-688 Krakow, Poland; jolanta.pyteraf@uj.edu.pl (J.P.); witold.jamroz@uj.edu.pl (W.J.); Joanna.szafraniec@uj.edu.pl (J.S.-S.); renata.jachowicz@uj.edu.pl (R.J.); 2 Department of Biophysics and Molecular Physics, Institute of Physics, University of Silesia, Uniwersytecka 4, 40-007 Katowice, Poland; daniel.kramarczyk@smcebi.edu.pl (D.K.); karolina.jurkiewicz@us.edu.pl (K.J.); justyna.knapik-kowalczuk@us.edu.pl (J.K.-K.); marian.paluch@us.edu.pl (M.P.); 3 Silesian Center for Education and Interdisciplinary Research, University of Silesia, 75 Pulku Piechoty 1a, 41-500 Chorzow, Poland; 4 Faculty of Physics and Applied Computer Science, AGH University of Science and Technology, Al. Mickiewicza 30, 30-059 Kraków, Poland; tarasiuk@agh.edu.pl (J.T.); wronski@fis.agh.edu.pl (S.W.)

**Keywords:** 3D printing, fused deposition modeling, hot-melt extrusion, solid dosage forms, immediate-release, sustained-release, ketoprofen

## Abstract

The flexibility of dose and dosage forms makes 3D printing a very interesting tool for personalized medicine, with fused deposition modeling being the most promising and intensively developed method. In our research, we analyzed how various types of disintegrants and drug loading in poly(vinyl alcohol)-based filaments affect their mechanical properties and printability. We also assessed the effect of drug dosage and tablet spatial structure on the dissolution profiles. Given that the development of a method that allows the production of dosage forms with different properties from a single drug-loaded filament is desirable, we developed a method of printing ketoprofen tablets with different dose and dissolution profiles from a single feedstock filament. We optimized the filament preparation by hot-melt extrusion and characterized them. Then, we printed single, bi-, and tri-layer tablets varying with dose, infill density, internal structure, and composition. We analyzed the reproducibility of a spatial structure, phase, and degree of molecular order of ketoprofen in the tablets, and the dissolution profiles. We have printed tablets with immediate- and sustained-release characteristics using one drug-loaded filament, which demonstrates that a single filament can serve as a versatile source for the manufacturing of tablets exhibiting various release characteristics.

## 1. Introduction

Additive manufacturing is forecast to become a significant element of the fourth industrial revolution leading to mass customization of medicine. Three-dimensional printing technologies allow the production of personalized fix-dose combinations with the desired release profile, which simplifies therapy and minimizes the risk of side effects related to under- or overdosing [1]. Additionally, in the case of printed dosage forms, their shape, color, or even flavor can be easily modified, which increases the attractiveness of therapy and affect patients’ compliance—the two aspects particularly important in the case of pediatric or geriatric groups [2]. Due to the high precision of 3D printing technologies, it is even possible to prepare Braille-encoded drug delivery systems for blind and partially sighted patients, to minimize the risk of mistake [3].

The term “3D printing” relates to the manufacturing of objects by extrusion or solidification of powders, semisolids, or liquids [4]. The extrusion-based 3D printing technologies can be divided into two main categories: methods relying on extruding semisolid formulations at room temperature (without melting materials) like a pressure-assisted microsyringe, and technologies based on extrusion of melted materials, including fused deposition modeling (FDM) [4,5]. These technologies, especially methods using melted polymers, are very promising 3D printing methods for preparing patient-specific medicines. It was confirmed by the recent approval of T19 for the Investigational New Drug (IND) program by the US Food and Drug Administration (FDA). T19 is a chronotherapeutic drug delivery system indicated for the treatment of rheumatoid arthritis, manufactured using the melt extrusion deposition technology developed by Triastek [6]. If it passes the clinical trials, it may become the second, after Spritam [7], approved 3D printed human drug product.

The FDM printing technique relies on depositing, in a controlled manner, a melt containing thermoplastic polymer and an active pharmaceutical ingredient (API). Numerous studies on the application of FDM technology in the manufacturing of drug delivery systems have been published confirming the versatility of that method. They include printing orodispersible films [8], mucoadhesive films [9,10], mini tablets [11], immediate [12,13], modified [14], or sustained-release [15], tablets and capsules, pH-responsive capsules [16], floating capsules and tablets [17,18,19], and capsules with a pulsating release profile [20]. Prior to printing, the FDM method requires the preparation of the feedstock material in the form of a filament, which is usually obtained in the hot-melt extrusion (HME) process [21]. In contrast to other printing methods, in the case of printing objects with simple structures such as tablets, the FDM printing method does not require any postprocessing steps such as drying or removing residual materials.

In contrast to the traditional production of tablets, consisting mainly of pressing a mixture of powders or granules, the relatively high temperature of HME and FDM processes can significantly affect the properties of the formulation ingredients. It can lead to physicochemical changes of the active substance, causing its amorphization [10,13,22] or formation of liquid crystals [23], which can improve the drug dissolution rate. However, it is also related to a significant limitation of the application of fused deposition modeling because the formulation cannot contain thermolabile substances. To minimize the exposure of the drug substance to high temperatures during the processes, polymers with low glass transition temperatures can be used as filament-forming substances [24]. A wide range of thermoplastic polymers can be used as filament-forming pharmaceutical-grade matrices including water-soluble polymers [18,20,23], pH-independent retarding agents [25,26], pH-responsive polymers [16,20,25], and others. The properties of the polymer, and in particular its water solubility, significantly affect the drug dissolution rate. Another way to decrease the processes temperature is to add plasticizers such as triethyl citrate [9,10,22], mannitol [22], sorbitol [27], and polyethylene glycol [22,28] into the formulation. An additional benefit of the use of plasticizers is the improvement of filament printability, which affects the process repeatability and increases the quality of printlets.

Other substances that can be added are colorants and disintegrants. They modify the drug dissolution rate and change the color of the printlets. However, due to the above-mentioned HME and FDM process conditions, the performance of some excipients may be different from that of a tablet of the same composition obtained by compression. The challenge is also to adjust the conditions of the HME process to prepare high-quality printable filaments, which affects the homogeneity of the printed dosage forms [29]. In our previous work, we showed the effectiveness of a 4% addition of crospovidone to accelerate the release of itraconazole from 3DP formulations [23]. Tablets containing superdisintegrants were also printed by Than and Titapiwatanakun. They optimized formulations containing theophylline with the addition of sodium starch glycolate to improve the drug dissolution rate. As a result, they printed immediate-release tablets containing up to 60% of API [30]. Moreover, Hussain et al. have proven the effectiveness of sodium starch glycolate and croscarmellose sodium in printlets containing captopril, made from hydroxypropyl cellulose [31]. Interestingly, in both cases, the disintegrant concentrations were higher compared to the amounts normally used in compressed tablets and were equal to 10 or 15% for sodium starch glycolate and 10% for croscarmellose.

Additional modification of the printlet can be achieved using a 3D printer equipped with two nozzles (dual-extrusion printing) or by co-extrusion of two materials using only one nozzle. In the first case, some parts of the tablet can be made of placebo filament, for example, to print the coating of the tablet [32,33]. The co-extrusion printing is based on simultaneous extrusion of two materials using only one nozzle, which leads to the preparation of a printlet with a complex structure at the level of a single layer. The co-extrusion of API-loaded filament and insoluble placebo filaments leads to further modification of drug release, as compared with extrusion of a single filament [34,35].

Among the main advantages of using 3D printing technologies in the manufacturing of drug delivery systems, the simplicity of dosage modification is highly appreciated. Two different approaches can be considered to apply dose personalization: (i) changing the amount of active ingredient in the filament and (ii) modifying the project by changing the dimensions or infill density of the tablet. Although the production of low-dose dosage forms does not cause many difficulties, an increase of the API content is usually associated with a significant decrease in printability; several works reporting the possibility of printing from filaments containing even 50–60% (*w*/*w*) of the API have been recently published [36,37,38]. Sadia et al. printed tablets using filaments containing from 2.5% to 50% *w*/*w* of hydrochlorothiazide using Eudragit PO with addition of triethyl acetate and tricalcium phosphate. All extruded filaments were printable [36]. In other work, Đuranović et al. prepared extended-release tablets using filaments containing up to 60% of paracetamol. As filament-forming matrices, two types of mixtures were tested: (i) polycaprolactone (PCL) with 10% arabic gum and (ii) polyethylene oxide (PEO) 200 K or PEO 100 K with 5% addition of arabic gum or Gelucire^®^ 44/14. Among the formulations containing 60% of API, only filament based on PCL and made from PEO 200 K with the addition of Gelucire^®^ 44/14 were printable. The authors also noted that printing with high drug-loaded filaments is difficult due to the clogging of the printer nozzle [37]. The 3DP prolonged-release tablets containing theophylline and metformin were reported by Verstraete et al. As filament-forming polymers, they used hydrophilic and hydrophobic thermoplastic urethanes containing 20%, 40%, or 60% of API, respectively. All formulations were printable, but as the API concentration increased, the surface of the filaments became rough [38].

The other method of dose modification relies on the reduction of the tablet infill density. It results in weight and dosage reduction, and an increase in the porosity, which accelerates API’s dissolution [23,24,26]. It should be noticed that both of the above-mentioned dose-adjusting methods can significantly affect the dissolution rate, which is essential in printing the customized drug delivery systems with desired release kinetics.

The main objective of this study was to conduct an in-depth analysis of the factors affecting the release of ketoprofen from 3DP tablets, which may be prepared on demand as a potential drug delivery system for personalized medicine. Types of disintegrants and drug loading in the polymer matrix made from poly(vinyl alcohol) were evaluated in order to obtain a universal filament composition exhibiting acceptable mechanical properties, good printability, and tunable dissolution performance. The effect of drug dosage and tablet spatial structure on the dissolution profile was also assessed. Our aim was to develop a high drug-loaded filament that can be used to produce immediate or sustained-release tablets, with the dissolution characteristics tuned by changing the design of 3D printed tablet. Ketoprofen, which belongs to Biopharmaceutical Classification System (BCS) class II [39], was selected as a model drug due to its poor solubility in water and availability in various dosage forms and doses up to 200 mg. We prepared filaments with various drug content (20%, 40%, or 50%) and disintegrants and optimized process parameters for each formulation. Then, we printed tablets that differed in composition, drug dosage, and internal structure to assess the suitability of prepared filaments for the preparation of the dosage forms with different properties. To obtain various drug release profiles, we selected the best filament composition and used it to prepare single, bilayer, and tri-layer tablets, and two formulations using co-extrusion 3D printing method.

## 2. Results and Discussion

### 2.1. Extrusion Process and Filament Characteristics

All hot-melt extrusion processes were optimized in order to obtain a repeatable filament diameter, preferably 1.75 mm. However, in the Voxelizer, the slicing software we used, it is possible to enter the actual diameter of the filament and adjust the project to fabricate repeatable printlets, despite the differences in filament diameter. Therefore, the main aim of the extrusion optimization was to produce filaments with a uniform diameter, not necessarily equal to 1.75 mm.

Different temperature profiles were set depending on the ketoprofen content. The higher the ketoprofen content, the lower the temperature of the extrusion, which is caused by the low melting temperature of ketoprofen equal to 94–97 °C [40]. The torque, which can be one of the limitations of the hot-melt extrusion technology, was in the range of 1.65 to 3.65 Nm, which is an acceptable level.

The data presented in Table 1 revealed slight deviations from the assumed ketoprofen content in the filaments. However, they were an acceptable range. Filament diameter was controlled during the optimization stage moreover, the diameter of the final filament was additionally measured manually, and the results are presented in Table 1. The desirable standard deviation (SD) value of the diameter is 50 µm. We were unable to achieve the filaments with such uniform dimensions for the formulation containing croscarmellose sodium (VSOL) and for the formulation with 50% ketoprofen content. However, for other API-loaded formulations, the deviations were even lower. Another important parameter in terms of 3D printing is the elasticity module, which was evaluated in the measurements of Young’s modulus in the stretching test. The tensile strength was not determined as the filaments were highly elastic and tended to slip away from the grips of the texture analyzer. All the filaments were characterized by appropriate mechanical properties for 3D printing, except for the one containing 50% of ketoprofen, i.e., F6, which was highly flexible and bent between the driving gear and the nozzle of the printhead. To increase the stiffness of the filament, it was stored in the desiccator, which led to the reduction of the plasticizing effect of water making printing possible. The Young’s modulus of the F6 filament after storage in the desiccator was equal to 381.81 ± 45.37 N/mm^2^, which was nearly 2-fold higher than for the same filament stored under ambient conditions. This shows the increase in the material stiffness, which is of crucial importance in terms of feeding the 3D printer.

### 2.2. 3D Printed Tablets

**Figure 1:**
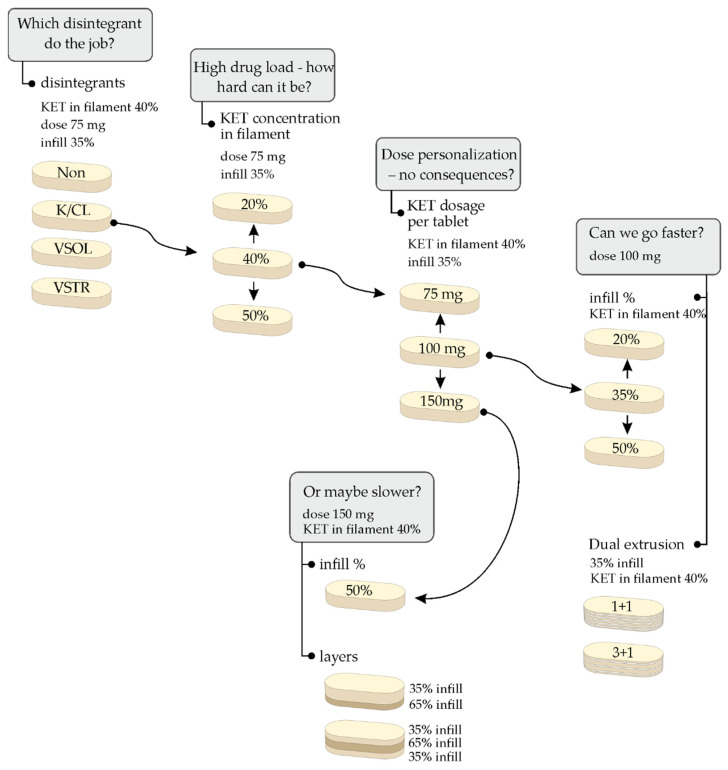
Schematic representation of the design of performed 3D printing experiments.

All tablets were printed based on a 10 mm × 20 mm oblong tablet model developed in 3D modeling software. The parameters of the printing process were adjusted to the composition of the filaments to achieve a continuous material deposition and to minimize differences in API dose. The filaments with different content of API (20%, 40%, and 50%) were used to obtain single-layer tablets with three infill densities (20%, 35%, 50%) and three doses (75 mg, 100 mg, 150 mg) as well as bi- and three-layer tablets with infill densities 35% and 65% and doses of 150 mg. In consequence, the height and the mass of the tablets were adapted and ranged from 1.50 mm to 3.90 mm and from 157 mg to 508 mg respectively, to achieve the predefined API dosage in tablets (Table 2). A schematic design of all stages of the experiment is presented in Figure 1.

The high homogeneity of the filaments’ diameter and high cohesiveness of the layers contributed to the repeatability of the printing process and minimized the variation of the tablets’ mass, which did not exceed the range of pharmacopeial requirements. The tablets were from light beige to beige in color and uniform in shape. For all formulations, both the extrusion and printing temperatures did not exceed 195 °C, i.e., laid below the ketoprofen decomposition temperature, which occurs at 235 °C [40].

### 2.3. X-ray Powder Diffraction (XRPD)

**Figure 2:**
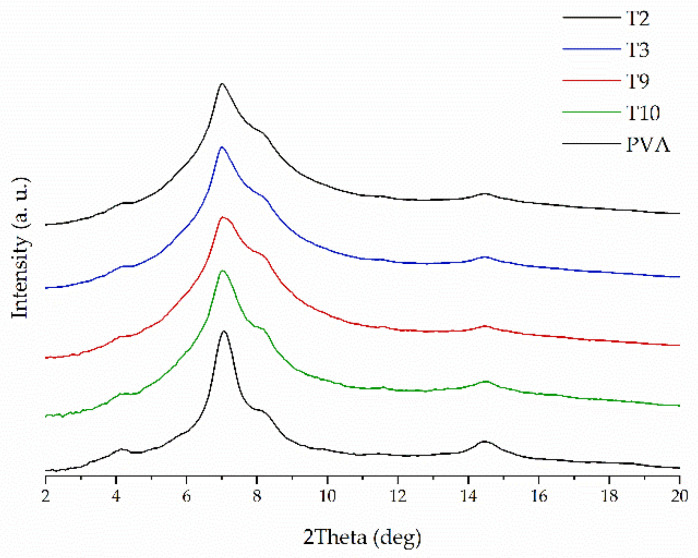
XRPD diffraction patterns of neat PVA and 3DP tablets containing 40% of KET with the addition of various disintegrants.

**Figure 3:**
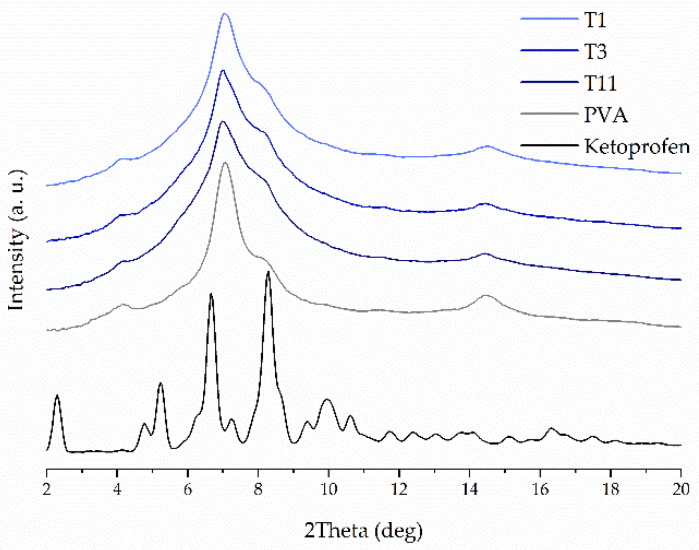
XRPD diffraction patterns of neat KET, neat PVA, and 3DP tablets made from filaments containing 20%, 40%, or 50% of KET with the addition of crospovidone.

**Figure 4:**
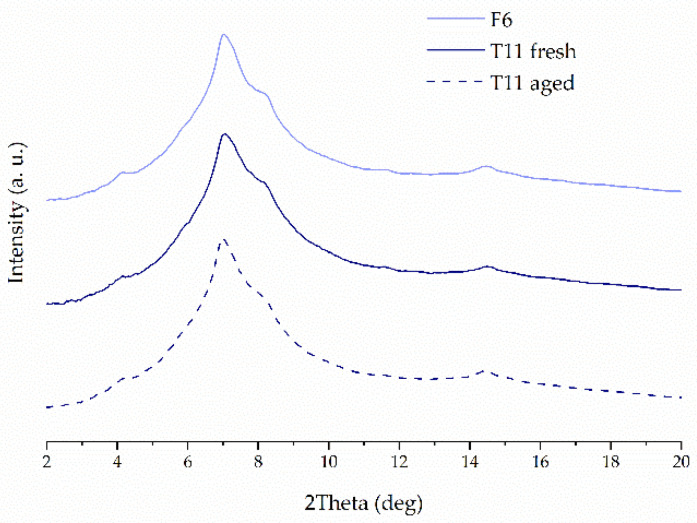
XRPD diffraction patterns of filament, fresh, and aged 3D printed tablets composed of 50% KET, crospovidone, and PVA.

The crystalline structure of raw compounds, filaments and tablets was studied using the X-ray diffractometry. Figure 2 shows representative diffractograms for 3DP tablets with 40% concentration of ketoprofen either with or without the addition of disintegrants. It can be clearly seen the XRD patterns resemble this one for pure PVA, which is the main component of the tablets. The diffractogram of pure PVA, with a number of broad but distinct peaks, suggests its semicrystalline nature. The maxima around 4°, 6°, 7°, and 8° are characteristic PVA (100), (001), (101), and (200) reflections, respectively, from monoclinic unit cell [41]. There are no signs of recrystallization of ketoprofen and other additives in the 3DP tablets that means ketoprofen is amorphous in the printed tablets. The measurements performed for tablets containing 20%, 40%, and 50% of ketoprofen confirmed that the API remains in amorphous phase, regardless of its concentration in the formulations. Figure 3 presents the representative XRD patterns collected for the 3DP tablets with K/CL additive. They demonstrate no evidence of ketoprofen recrystallization. In turn, in Figure 4 the XRD data for freshly prepared T11 tablets are compared with the data for the tablets stored by two weeks (aged tablets) and with the data for the filament of the same composition. There are no distinct differences in the atomic-scale structure of these three samples, indicating the physical stability of the tablets over two weeks of storage.

### 2.4. Differential Scanning Calorimetry

**Figure 5:**
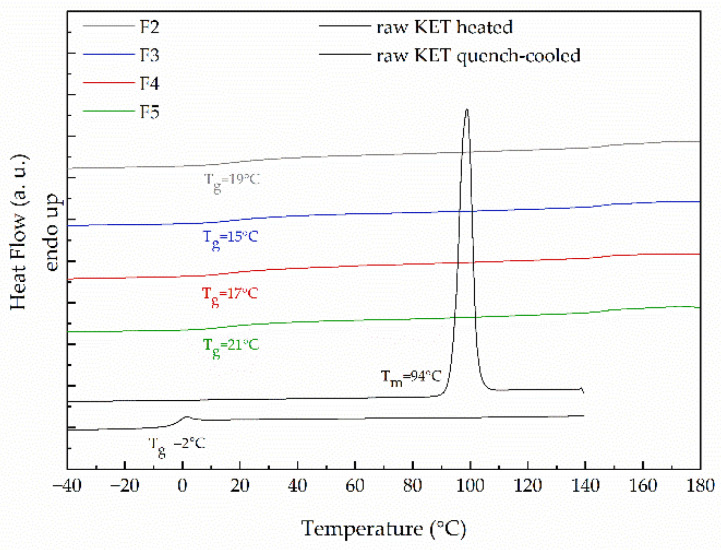
DSC thermograms of raw KET and filaments containing 40% of KET with the addition of various disintegrants.

Differential scanning calorimetry has been applied to study the thermal properties of the obtained drug–polymer systems and its physical stability. To investigate how the applied excipients affect the thermal properties of ketoprofen, raw KET and each type of filament were heated within the temperature range from −40 °C to 180 °C at a heating rate equal to 10 °C/min. The analysis of the thermogram collected for raw KET reveals the existence of a well-resolved endothermic peak corresponding to ketoprofen melting (Figure 5). The onset of the peak indicates that the melting point of raw KET is equal to 94 °C, which stays in agreement with Tiţa et al. [40]. Since the drug is characterized by very low glass transition temperature (T_g_ below 0 °C) [42], its amorphization and physical stability of the molecularly disordered state are realized as challenging. The value of glass transition temperature measured for raw, quench-cooled ketoprofen is equal to −2 °C. After the extrusion process, the drug undergoes amorphization as evidenced by the presence of glass transition event in the vicinity of 15–21 °C (selected thermograms presented in Figure 5). Given that the PVA has a higher glass transition temperature than ketoprofen (T_gPVA_ = 40–45 °C) [43], and its content in the filament compositions varies between 46–76%, its antiplasticizing effect on the API is noticed as an increase in KET T_g_ in the filaments. The formulation containing 40% of KET and 60% of PVA exhibits glass transition at 19 °C. The addition of other excipients leads to shifts in T_g_s towards lower temperatures, which may result from the sorption of small quantities of water. However, those changes are no bigger than several degrees, which indicates that at room temperature all the filaments are elastic and do not differ much from each other.

**Figure 6:**
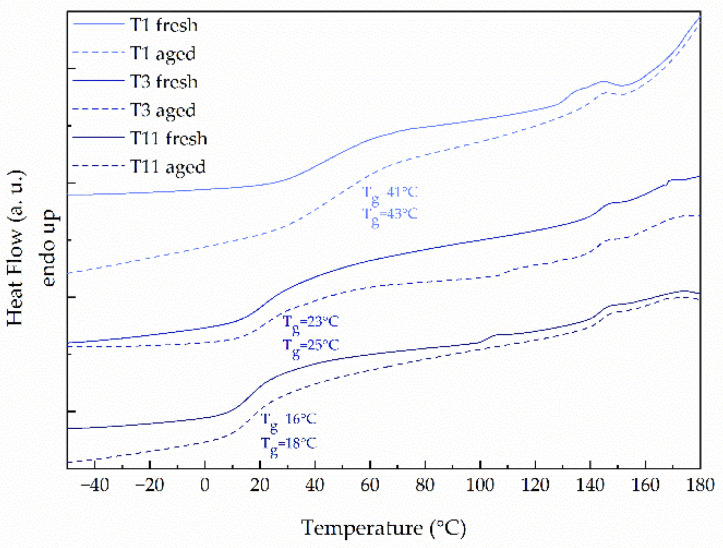
DSC thermograms of fresh and aged 3DP tablets made from filaments containing 20%, 40%, or 50% of KET with the addition of crospovidone.

After the second heating and cooling cycle, which occurred during the 3D printing, the drug exists in an amorphous state in all the examined samples. The results presented in Figure 6 reveal that the glass transition temperature of tablets rises slightly in comparison with the corresponding filament. Since the amorphous substances are physically unstable, we examined the effect of storage of printed tablets using DSC. We did not observe any signs of instability given by the decrease in T_g_ or recrystallization, which indicates that the tablets were physically stable after storage, despite the drug content in the formulation. What is also noticeable in Figure 6 is that the T_g_ of tablets decreases with an increase in the content of ketoprofen in the formulation, which is expectable and related to the low glass transition temperature of raw ketoprofen.

### 2.5. FTIR Spectroscopy

**Figure 7:**
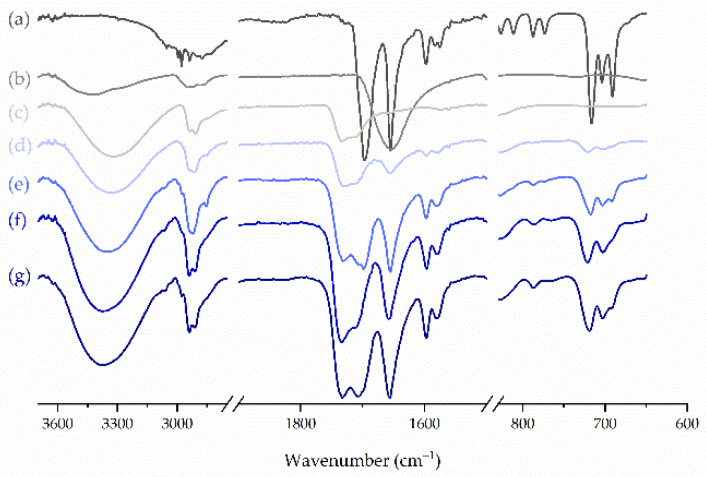
Selected FTIR spectra of investigated systems: (**a**) ketoprofen, (**b**) Kollidon^®^CL, (**c**) PVA, (**d**) F1, (**e**) F3, (**f**) T3, (**g**) F6.

FTIR spectroscopy was applied to characterize the molecular structure of the compounds and to analyze the interactions between ketoprofen and other components of either filaments or printed tablets. The analysis of the spectrum of raw ketoprofen revealed the presence of intensive peaks corresponding to stretching and bending vibrations of its functional groups (Figure 7). The intense, sharp bands with maxima at 1697 cm^−1^ and 1695 cm^−1^ result from the stretching vibrations of the carbonyl group in the dimeric carboxylic acid, and the ketonic group, respectively [44]. Low-intensity bands in the vicinity of 3000 cm^−1^ correspond to the antisymmetric vibrations of CH_3_ groups, while the band at 3296 cm^−1^ represents the stretching vibrations of the H-bonded hydroxyl group, which confirms the presence of dimeric entities of ketoprofen in the condensed phase [45]. The fingerprint region of crystalline ketoprofen shows the presence of an intensive triplet with maxima at 716 cm^−1^, 704 cm^−1^, and 691 cm^−1^, which correspond to C-H and C-*O*-H out-of-plane bending vibrations. After extrusion and 3D printing, the intensity of these vibrations decreased, the band broadened and became a doublet in the case of tablets, which may indicate the formation of an amorphous phase. The aforementioned effects were more pronounced for tablets and enhanced for the samples containing a lower amount of ketoprofen, more likely due to the lower content of the crystalline phase.

In the case of PVA, which represents the largest part in every sample, the FTIR spectrum shows a broad band with a maximum at 3331 cm^−1^, which is assigned to the stretching vibration of the O-H group involved in the formation of hydrogen bonds (Figure 7). The intensive band in the vicinity of 2900 cm^−1^ reflects the stretching of C-H groups in alkyl chains, and the one at 1734 cm^−1^ comes from the absorption of residual acetate groups resulted from the incomplete hydrolysis of polyvinyl acetate [46]. Given the lack of proton acceptor in the PVA molecule, no interactions with ketoprofen were expected. This assumption was consistent with the analysis of the spectra of both filaments and 3D printed tablets, which revealed that they are a summation of the spectra of the polymer with the crystalline and amorphous drug. Similarly, no interactions were detected between ketoprofen and any other excipient.

### 2.6. Micro-Computed Tomography

A single tablet from T4, T5, T6, T12, and T13 batches was chosen for µCT analysis to investigate the internal structure of the tablets and print quality. All the analyzed tablets were printed with the best selected F3 filament consisting of 40% of ketoprofen and 4% of crospovidone as a disintegrant. The tablets were accurately weighed and measured while the volume, unfolded surface area, and material volume fraction that corresponds to the ratio of the material to the voids in the cuboid inside the scanned object were calculated based on microCT scans. Additionally, average printing path dimensions were determined for single-layer tablets (T4, T5, and T6). Detailed characteristics of the analyzed tablets are presented in Table 3.

**Figure 8:**
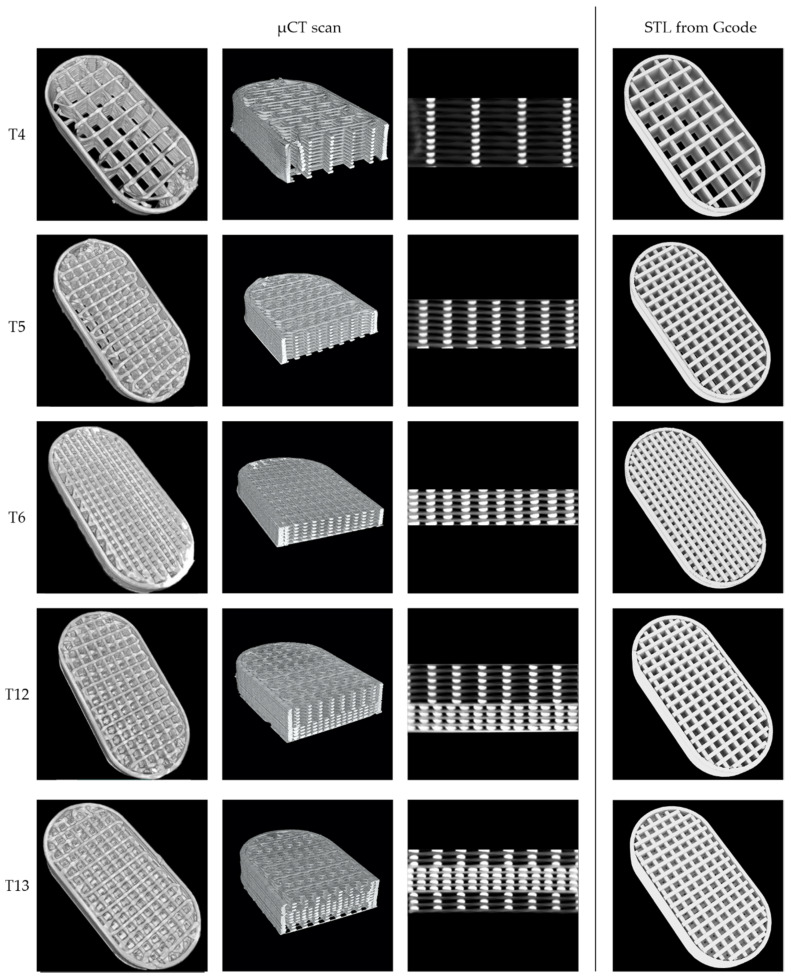
MicroCT scans and 3D design of the single-layer (T4, T5, T6), bilayer (T12), and trilayer (T13) tablets.

All the single-layer tablets, namely T4, T5, and T6, have a similar length and width, which results from the same tablet design. The differences in the height of the tablets are the effect of various infill densities, i.e., 20%, 35%, and 50% for T4, T5, and T6, respectively, introduced to get 100 mg of ketoprofen in each tablet. The calculated volumes of the tablets scaffolds are exactly the same, which confirms the high reproducibility of the 3D printing process (Table 4). The values of the total surface area might seem to be unexpected because the surface area of 20% infill tablets was lower than for 35% infill. However, the surface area values calculated by 3D design software, i.e., Blender and Photo Studio showed the same relationship. All the values calculated based on the 3D model are slightly higher than those measured based on µCT scans, which may result from small defects of the tablet structure, visible especially in 20% infilled tablets (Figure 8). The differences between the calculated and measured values decrease with the increase in the infill density, which also confirms that the differences were caused by the defects as with higher density of infill the number of defects is reduced. The fraction parameter describes the resultant value of the infill in the printed tablet and they are close to the assumed tablets project. A lower infill density causes the more distant deposition of infill lines and creates tablets with larger pores, leading to a decrease in the fraction parameter.

The analysis of the printing path height showed that for tablets with lower infill densities the printing path is higher than the project assumes. The height of the printing path for the T4 and T5 was 0.19 mm, while for T6 printed with 50% infill density it was equal to 0.16 mm, and it was closer to the assumed 0.15 mm. The differences in the printing path width were also noticeable. The theoretical width was set to 0.4 mm, while the resulting width was in the range of 0.38–0.42 mm. These discrepancies can be attributed to the Barus effect, which is related to the material swelling after being extruded through the printer nozzle. The Barus effect intensity depends on the process parameters and should be the same for all printed tablets as they were printed at the same temperature and speed. Additionally, the T6 tablets, which had the highest infill density, were characterized by the flattened printed layers, while T4 with a smaller infill density had narrower and higher paths. This, in turn, is caused by the impact of the mass of the successively printed layers, which is the most visible in cross-sections of the tablets. Both above-mentioned factors could contribute to the fact that the print paths were narrower and higher for the T4 tablets and wider and lower for the T6 tablets (Table 3 and Figure 8).

The different distribution of layers within the design of bilayer and trilayer tablets resulted in slight differences between the volumes of scanned objects. However, the difference is lower than 3% so it can be also attributed to the limited precision of the method. The T12 and T13 tablets have the same number of layers with 35% and 65% infill, so their height and calculated surface are almost the same.

### 2.7. Influence of Tablets Formulation on the Dissolution Profile

Disintegrants are widely used in pharmaceutical technology. They promote water penetration through solid dosage form by different mechanisms and, as a result, impairs tablet structure. Thus, the first question we asked was:

*Which disintegrant improves the dissolution of ketoprofen the most?*

Tablets containing 75 mg of the active ingredient with 35% infill, were fabricated using filaments of 40% concentration of ketoprofen, poly(vinyl alcohol), and disintegrants characterized by various disintegration mechanisms. Formulations containing crospovidone, which disintegrates tablets by swelling without gel formation [47], croscarmellose sodium, acting by causing a wicking effect [48], and sodium starch glycolate with a rapid and high degree of swelling [49] were tested.

**Figure 9:**
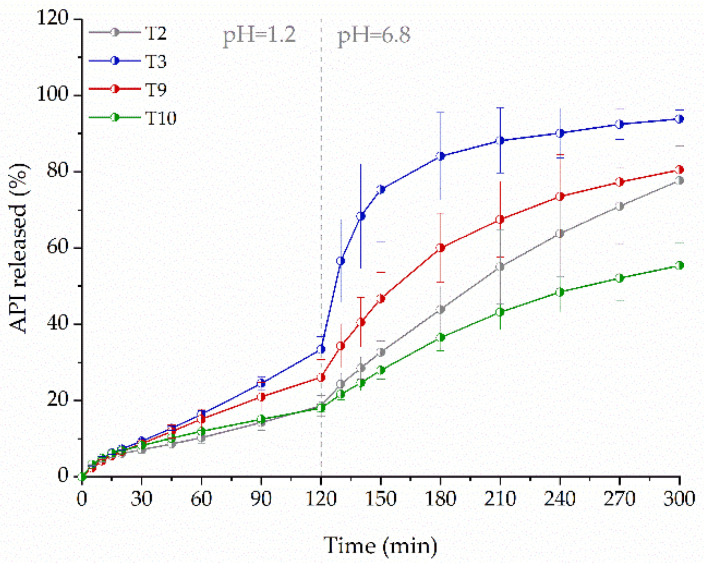
The influence of the disintegrants on dissolution profiles of ketoprofen from 3DP tablets (infill density equal to 35%, 40% of drug loading, dosage 75 mg).

The dissolution profiles in Figure 9 show that the presence of disintegrants increases the concentration of dissolved drug. The best result was obtained for tablets containing crospovidone (T3). After two hours, the amount of released drug was 1.8 times higher than from tablets without the disintegrant (T2). After three hours, the amount of released drug was almost twice as high. Croscarmellose sodium (T9) turned out to be a less effective disintegrant as compared to crospovidone (T3), the amount of released ketoprofen after three hours did not exceed 1.5 times the amount released from the formulation without disintegrants (T2). Although the mechanism of action of sodium starch glycolate, like that of crospovidone, is based on swelling, it led to a slower release of ketoprofen from the printed tablets (T10). Finally, the differences in the disintegrant particle properties and their swelling capacity affected drug dissolution [50].

Ketoprofen is used in different strengths, from 12.5 mg to 200 mg, which requires high drug load filament to prepare dosage form, with acceptable size. Thus, we decided to verify:

*What is the highest possible ketoprofen load in the filament?*

**Figure 10:**
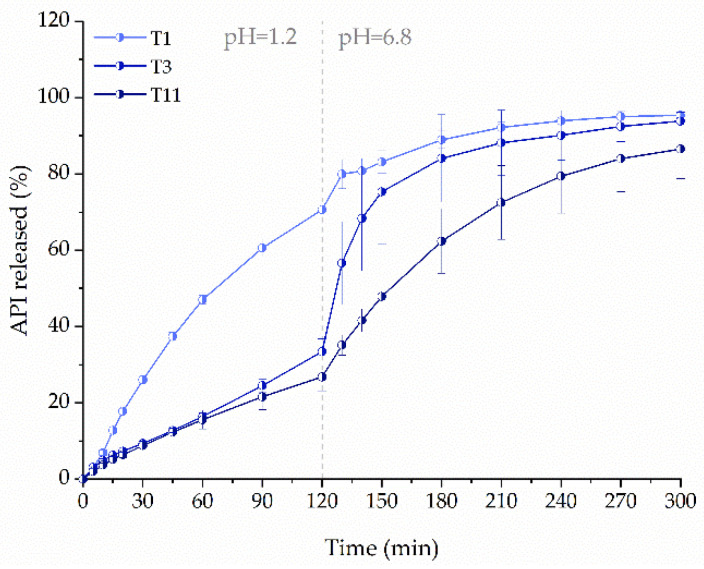
The influence of drug loading in filaments on dissolution profiles of ketoprofen from 3DP tablets (infill density equal to 35%, dosage 75 mg).

Although we succeeded in the preparation of the filament with 50% drug content, problems related to its high elasticity occurred. To evaluate the effect of ketoprofen content on its release, we compared tablets with 75 mg dose printed from the filaments with 20% (F1), 40% (F3), or 50% (F6) content of ketoprofen and 4% of crospovidone as a disintegrant. As the active substance content in filament increased, the tablet height was reduced to maintain the same dosage. Despite the reduction of tablet vertical dimension, the amount of released substance from tablets decreased (Figure 10). After two hours, the amount of ketoprofen released from T1 was more than two times higher than from the tablets with a higher drug load, which is related to the highest content of PVA and its solubilizing effect on ketoprofen in the acidic dissolution media. Additionally, the gradual disintegration of T1 tablets was observed during the dissolution, whereas T3 and T11 tablets were converted to shapeless swollen mass, which caused a diffusion barrier reducing the dissolution rate. In the acidic phase, the dissolution profiles of ketoprofen from tablets T3 and T11 were similar. However, after three hours, in the buffer phase, over 84% and 62% of the API was released from T3 and T11 formulations, respectively.

It is well known that 3D printing is recognized as a method for preparation of tailor-made medicines. Especially, flexibility in geometry and API dosage modification are usually highlighted. Therefore, we asked the question:

*Dose personalization with no consequences?*

**Figure 11:**
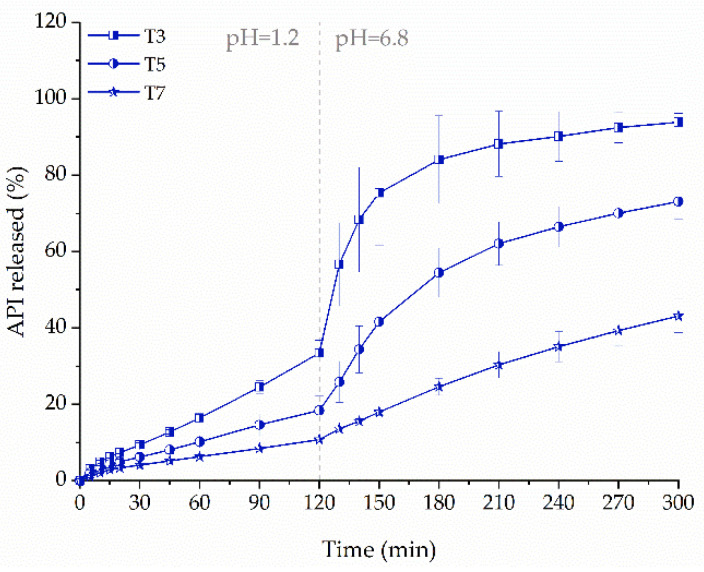
The influence of dosage on dissolution profiles of ketoprofen from 3DP tablets made from F3 filament (infill density equal to 35%).

We compared ketoprofen release profiles from tablets containing 75 mg (T3), 100 mg (T5), and 150 mg (T7) of the API and 35% infill, which were prepared from F3 filament. The increase in tablet vertical size, which was necessary to print tablets with higher ketoprofen dose, resulted in a decrease in the amount of released API (Figure 11). In both phases, the amount of released drug was approximately three times less from T7 in comparison with T3 formulation.

Ketoprofen is used in either immediate-release (IR) or sustained-release (SR) solid dosage forms. The dose of 100 mg of ketoprofen is recognized as the highest dose for IR tablets. Thus, we wonder:

*Can we go faster and improve dissolution rate of ketoprofen from IR tablets?*

We focused on the modifications of 100 mg ketoprofen tablets to obtain a dissolution profile similar to IR tablets. It was conducted by changing the infill of the tablets.

**Figure 12:**
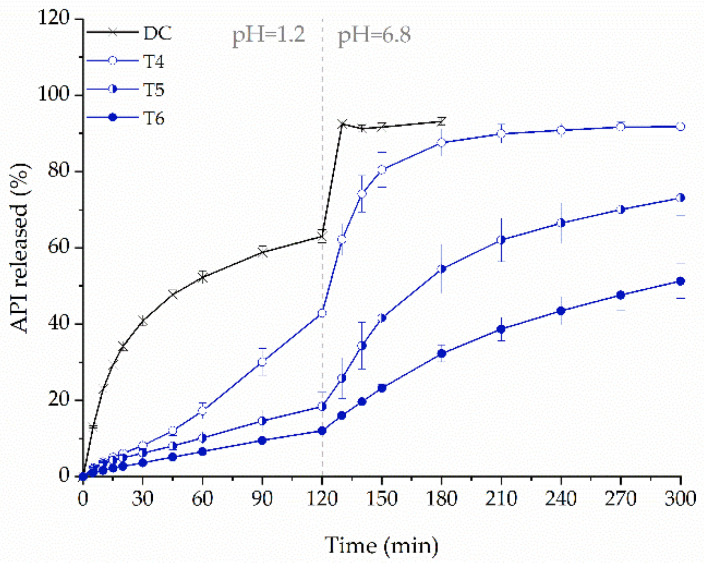
The influence of infill density on dissolution profiles of ketoprofen from directly compressed (DC) tablets and printlets made from F3 filament (dosage equal to 100 mg).

We compared tablets containing 100 mg API, prepared from F3 filament, printed with 20% (T4), 35% (T5), and 50% (T6) infill density. Decreasing the infill density caused a less frequent deposition of the molten formulation by the printhead, and printing of more porous tablets, leading to an increased release of ketoprofen (Figure 12). After two hours, the amount of dissolved drug from tablets T4 was 2.3 and 3.3-fold greater than from T5 and T6, respectively, and after five hours, 1.8 and 1.3-fold greater, respectively. It clearly indicates that the improvement in the dissolution rate of ketoprofen was achieved. For comparison purposes, the 100 mg IR tablets were prepared by direct compression (DC). After two hours of the study, the amount of API released from DC tablet was still 1.5 times higher than from 3D printed T4 tablets. In addition, the use of such a low infill density increased the size of the tablet, which may adversely affect the patient’s compliance. It raises a question of what else can be done to improve the dissolution rate.

The necessity of the application of different filaments with various API content to obtain desired dissolution profile is inconvenient and associated with increased manufacturing costs. The low drug-loading of filament, as well as low infill density of the tablet, lead to an excessive size of a tablet with a high dosage of API. The versatility of the FDM 3D printing method allows the preparation of dosage forms with different properties during a single technological process from one kind of feedstock filament. Therefore, we asked another question:

*Is further improvement of the dissolution possible?*

**Figure 13:**
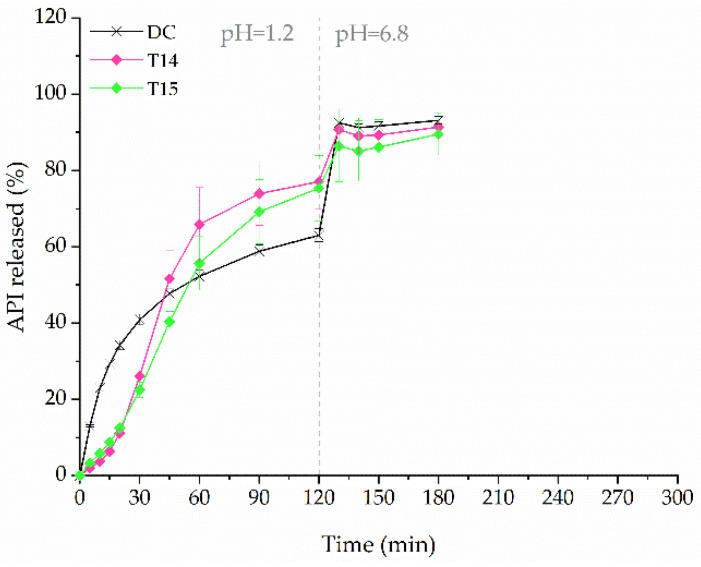
The dissolution profiles of ketoprofen from directly compressed (DC) tablets and co-extruded 3DP formulations (dosage equal to 100 mg).

In our previous study, we investigated the 3D printing method by means of filament co-extrusion of drug-loaded filament and insoluble filament to obtain sustained-release dosage forms, from which the release profile of API can be changed “on demand” [34]. In the present study, we adapted this method to improve the dissolution rate of ketoprofen from tablets printed using the PVA-based filament containing 40% of the drug (F3) and water-soluble Kollicoat^®^ IR-based placebo filament. Those filaments were simultaneously driven to the printhead. As a result, tablets T14 and T15 were formed, composed of filaments mixture containing either 50% or 25% of the placebo filament, encoded as 1 + 1 and 3 + 1 samples, respectively. The disruption of the PVA matrix by Kollicoat^®^ IR influenced the dissolution profile. After two hours, the amount of ketoprofen released from the tablets obtained by co-extrusion was 1.2 times higher than from the DC tablets (Figure 13). The increase in released ketoprofen is visible even for the T15 formulation, which contains only a 25% addition of placebo filament, which is not associated with a large increase in the dimensions of the tablet.

Based on the dissolution results it can be stated that high drug load in filament, as well as dose enhancement and increase of tablet infill, cause a decrease in the amount of ketoprofen released from tablets. Thus, the last question we asked:

*Whether the 3D printed tablets with ketoprofen can be used as a modified-release dosage form?*

**Figure 14:**
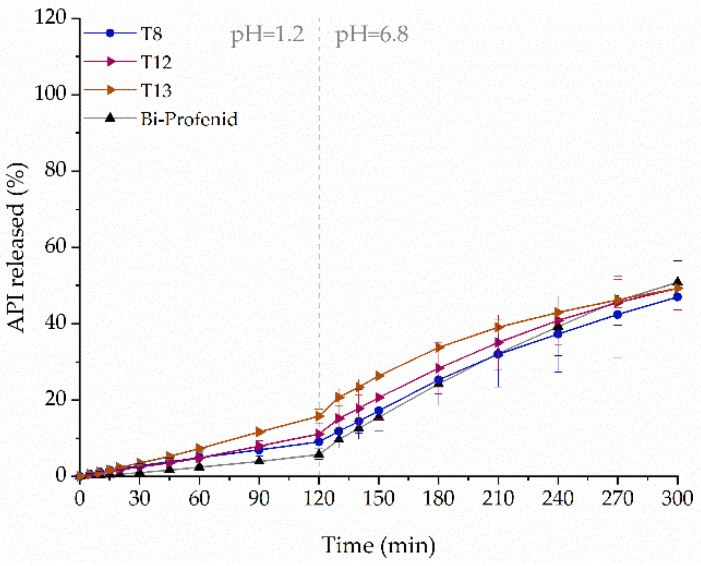
The dissolution profiles of ketoprofen from sustained-release formulations: Bi-Profenid and various 3DP tablets made from F3 filament (dosage equal to 150 mg).

For comparison purposes, commercially available Bi-Profenid tablets were used. It is a bi-layer tablet, and each layer contains 75 mg of the API. We began with printing a simple single-layer tablet T8 with 150 mg of ketoprofen with 50% infill, as it was earlier confirmed that higher infill densities cause slower API release. As the amount of dissolved ketoprofen from T8 tablets was higher in simulated gastric fluid and lower in simulated intestinal fluid in comparison to Bi-Profenid tablets, we decided to modify the structure of the tablets. To mimic the structure of the reference tablets, bi-layer (T12) and additionally tri-layer (T13) tablets were printed with compartments having smaller (35%) and higher (65%) infill density (Table 2 and Figure 1). The introduced structure modification led to the increased dissolution of ketoprofen in both phases of the dissolution test. The dissolution profiles of all printed tablets were similar to the reference; however, the smallest differences can be noticed for single-layer T8 tablets (Figure 14).

## 3. Materials and Methods

### 3.1. Materials

Ketoprofen (KET, 2-(3-benzoylphenyl)propanoic acid, 99.4%, Hangzhou Hyper Chemicals Limited, Hangzhou, China) was used as a model drug substance. Poly(vinyl alcohol) (PVA, Parteck^®^ MXP, Merck^®^-KGaA, Darmstadt, Germany) and polyvinyl alcohol/polyethylene glycol graft copolymer (KIR, Kollicoat^®^ IR, BASF^®^, Ludwigshafen, Germany) were used as filament forming polymers. Crospovidone (K/CL, Kollidon^®^ CL, BASF^®^, Ludwigshafen, Germany), croscarmellose sodium (VSOL, Vivasol^®^), and sodium starch glycolate (VSTR, Vivastar^®^) both form JRS PHARMA GmbH & Co. KG (Rosenberg, Germany) were used as disintegrants. Mannitol (Avantor^®^ Performance Materials Poland S.A., Gliwice, Poland) was used as a plasticizer. Silicified microcrystalline cellulose (Prosolv^®^ SMCC HD 90, JRS PHARMA GmbH & Co. KG, Rosenberg, Germany), lactose DC (SuperTab^®^ 40LL, DFE Pharma, Goch, Germany), talc (Fagron^®^, Kraków, Poland) and magnesium stearate (Avantor^®^ Performance Materials Poland S.A., Gliwice, Poland) were added to tablets prepared by direct compression. Bi-Profenid, conventional ketoprofen 150 mg sustained-release tablets (Sanofi-Aventis France, Paris, France), were used for comparison purposes. Hydrochloric acid and trisodium phosphate dodecahydrate, both from Merck^®^ KGaA (Darmstadt, Germany), and potassium chloride (Avantor^®^ Performance Materials Poland S.A., Gliwice, Poland), were used as dissolution media ingredients. Water used in all experiments was produced by Elix 15UV Essential reversed osmosis system (Merck^®^ KGaA, Darmstadt, Germany).

### 3.2. Filaments Manufacturing by Hot-Melt Extrusion

**Figure 15:**
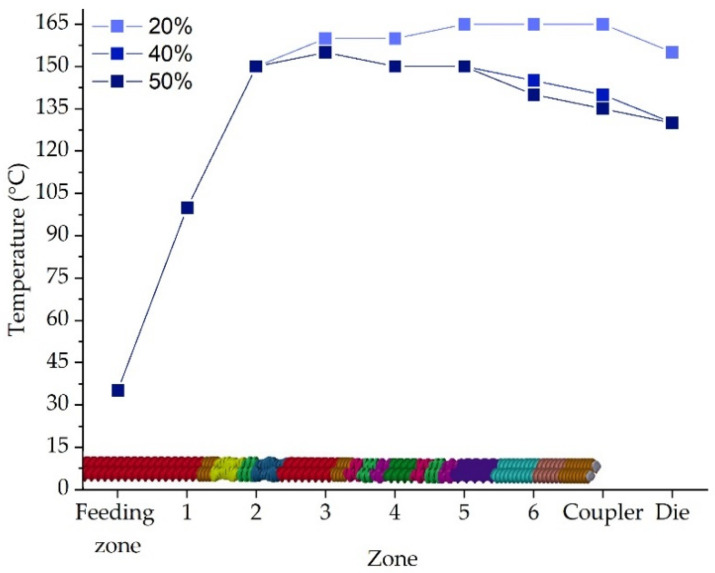
Screw design and temperature profile for formulations with different ketoprofen concentrations.

The filaments were prepared by means of hot-melt extrusion using a 12-mm co-rotating twin-screw extruder (RES-2P/12A Explorer, Zamak Mercator^®^, Skawina, Poland) with a barrel length of 40D. The extrusion line was equipped with a gravimetric feeder (MCPOWDER^®^ Movacolor^®^, Sneek, The Netherlands), an air-cooled conveying belt (Zamak Mercator^®^, Skawina, Poland), and a two-dimensional laser diameter gauge (LDM25XY, Mercury-Tech Co., Ltd., Zhengzhou, China). The mixtures of ketoprofen, poly(vinyl alcohol), and disintegrants were prepared by geometric dilution and extruded. Drug-free filament composed of Kollicoat^®^ IR and mannitol was prepared as a modifying filament for co-extrusion 3D printing experiment. The compositions of the filaments are presented in Table 5. The feeding rate and the screw speed were maintained at the same level for all extrusion experiments and were equal to 50 g/min and 45 rpm, respectively. The mixtures were extruded through a 1.75 mm die at different temperature profiles matched to the composition. The optimized barrel temperature profiles are presented in Figure 15.

### 3.3. Design and 3D Printing of Tablets

Oblong tablets (10 mm wide and 20 mm long) were designed using Blender 2.90 software (Blender Foundation, Amsterdam, The Netherlands). The height of the tablets, which was related to the number of 3D printed layers, ranged from 1.50 to 3.90 mm to achieve the targeted ketoprofen dosage. Voxelizer^®^ slicing software (version 1.4.18, ZMorph^®^, Wroclaw, Poland) was used as a slicing software to adjust the printing parameters and prepare gcodes for the 3D printing process. All formulations were printed in the same conditions including single layer height equal to 0.15 mm, printing speed of 15 mm/s, single outline, and rectilinear infill with 20, 35, or 50% density. Each formulation was extruded through a 0.4 mm nozzle at the temperature ranged from 175 to 185 °C, depending on the composition of the filament. When changing the formulation, the filament diameter set in the slicer was adjusted to the actual filament diameter, to achieve a homogeneous material deposition and to minimize differences in tablet weight. The tablets were printed by an FDM ZMorph^®^ 2.0 S personal fabricator (Wroclaw, Poland) equipped with a 1.75 mm commercially available printhead.

To evaluate the effect of the excipients on the release profile, tablets with 35% of infill, containing 75 mg of ketoprofen were printed using filaments containing 40% of API with the addition of various disintegrates. Next, to analyze the effect of infill density, drug dosage and API content on the dissolution rate, tablets with a 20%, 35%, or 50% infill density, containing 75, 100, or 150 mg of API were printed using filaments containing 20%, 40%, and 50% of ketoprofen with the addition of Kollidon^®^ CL.

The effect of the spatial structure of the tablets was also examined to prepare sustained-release dosage forms. Two types of formulations containing 150 mg of the API made of F3 filament were printed: (i) bi-layer tablets made of 12 layers with 35% infill and 9 layers with 65% infill, and (ii) three-layer tablets composed of two 6-layer parts with 35% infill separated by a 9-layer part with 65% infill.

Additionally, DualPro 1.75 mm printer toolhead was used to prepare immediate-release tablets with 35% rectilinear infill by co-extrusion of a F3 filament with a water-soluble placebo filament (KIR_M, PF). The parameters of printing process were the same as previously described. However, the temperature necessary for printing with the addition of the placebo filament was higher, equal to 192 °C. The height of the tablets was 5.40 mm or 4.05 mm, depending on the drug-loaded vs. placebo filament ratio. Each tablet contained 100 mg of ketoprofen.

### 3.4. Preparation of Directly Compressed Tablets (DC Tablets)

For comparison purposes, round tablets with a 10 mm of diameter, containing 100 mg of ketoprofen and Prosolv, 70 mg of lactose, 15 mg of crospovidone, and 15 mg of talc and magnesium stearate mixture (9:1 *w*/*w*) were manufactured using a Korsch^®^ EK0 single-punch tablet press (Berlin, Germany).

### 3.5. Filament Properties’ Evaluation

The filament diameter uniformity was measured on-line during the extrusion process using a two-dimensional laser diameter gauge (LDM25XY, Mercury-Tech Co., Ltd., Zhengzhou, China) and an off-line Mitutoyo^®^ micrometer screw (Kawasaki, Japan). Mechanical properties of the filaments were evaluated in a stretching test using an EZ-SX tensile tester (Shimadzu^®^, Kioto, Japan) equipped with a 500 N load cell. Six randomly taken filament pieces from each formulation were tested by placing between two tensile tester grips and were stretched with 1000 mm/min speed up to breakage. The gauge length was equal to 100 mm, and the precisely measured diameter of the tested specimens was included. The Young’s modulus values were calculated in the elastic behavior region using a Trapezium X software (Ver. 1.3.0 Shimadzu^®^, Kioto, Japan).

### 3.6. Determination of Ketoprofen Content in the Filaments

Three randomly selected and accurately weighed pieces of filament were placed in conical flasks filled with 50 mL of a phosphate buffer solution pH = 6.8 and shaken for 24 h using a Memmert^®^ water bath (WNB 22, Schwabach, Germany). The samples were filtered through CHROMAFIL^®^ Xtra CA-45/25 syringe filters and the API concentration was assayed spectrophotometrically at λ = 260 nm using a Shimadzu^®^ UV-1800 spectrophotometer (Kioto, Japan). The specificity of the analytical method was verified, there was no sign of interference between the drug and excipients at the analytical wavelength. The linearity was confirmed in the range from 5 to 25 µg/mL (R^2^ = 0.99999).

### 3.7. FTIR Spectroscopy

The vibrational spectra of raw compounds, filaments, and 3D printed tablets were collected using an attenuated total reflectance mode on diamond as an ATR crystal. A Nicolet iS10 spectrometer (Thermo Fisher Scientific, Waltham, MA, USA) equipped with a Smart iTR™ sampling accessory was used. The spectra (128 scans for each sample) were collected within the range 600–4000 cm^−1^ with 4 cm^−1^ resolution.

### 3.8. Micro-Computed Tomography

The X-ray measurements were performed using a “Nanotom 180S” device produced by GE Sensing & Inspection Technologies phoenix|X-ray GmbH (Wunstorf, Germany). The machine is equipped with nanofocus X-ray tube with maximum 180 kV voltage. The tomograms were registered on a Hamamatsu 2300 × 2300 pixel detector. During the measurement, the tungsten target was used. The polychromatic beam was not filtered. The working parameters of X-ray tube were as follows: intensity and voltage equal to 80 µA and 100 kV, respectively. The number of taken projections was equal to 1800, three integrations for each exposition and single dead frame registration. The total time of 360° measurement was around 90 min. The reconstruction of measured objects was done with the aid of proprietary software datosX ver. 2.1.0 (GE Sensing & Inspection Technologies phoenix|X-ray GmbH, Wunstorf, Germany)with use of Feldkamp algorithm for cone beam X-ray CT [51]. The final resolution of reconstructed object was 10 µm. The post reconstruction data treatment was performed using VGStudio Max 2.1 and some data analyses were performed using a FiJi tool with installed BoneJ plugin [52].

For comparison purposes, gcode files used to print tablets were converted into stl files using a Voxelizer software. Next, the volumes and surface areas of tablets were calculated using two methods: in Photocentric Studio 3D slicing software (version 1.0.2.9, Photocentric Ltd., Peterborough, UK), and using Blender 3D computer graphics software. Calculated results were compared with the values measured by microCT.

### 3.9. Differential Scanning Calorimetry (DSC)

Thermodynamic properties were examined using a DSC 1 STAR^e^ System (Mettler-Toledo^®^, Greifensee, Switzerland). The apparatus was equipped with an HSS8 ceramic sensor (heat flux sensor with 120 thermocouples) and liquid nitrogen cooling accessory. The calibration of temperature and enthalpy was performed using zinc and indium standards. The samples were measured in pinned aluminum pans of volume equal to 40 µL in nitrogen atmosphere; gas flow was maintained at 60 mL/min level. The heating rate was equal to 10 °C/min. Melting points of the samples were determined as an onset of the peaks, and glass transition temperatures were determined as the midpoint of the heat capacity increment.

### 3.10. X-ray Powder Diffraction (XRPD)

XRD patterns were collected using a Denki^®^ D/MAX Rapid II-R diffractometer (Rigaku, Tokyo, Japan). The apparatus was equipped with a rotating Ag anode, a graphite (002) monochromator, and an image plate detector in the Debye–Scherrer geometry. The wavelength of the incident beam, λKα, was 0.5608 Å. The measurements were performed at room temperature. The samples were pulverized and measured in borosilicate glass capillaries having 1.5 mm in diameter and 0.01 mm of wall thickness. The beamwidth at the sample was 0.3 mm. The collected two-dimensional diffraction patterns were corrected for background from the empty capillary and converted into one-dimensional functions of scattering intensity versus the scattering angle. At the end, the diffractograms were normalized to the intensity of the main peak.

### 3.11. Dissolution Studies

The release of ketoprofen from 3D printed tablets was performed using a USP-II apparatus (Hanson Research SR8 Plus, Chatsworth, CA, USA) equipped with the autosampler. Ketoprofen solubility, as a propionic acid derivative is strongly related to the pH of the solute. Therefore, two-phase dissolution method was applied. For the first 120 min, 750 mL of 0.1 M HCl with the addition of potassium chloride at pH = 1.2 was used to mimic gastric conditions. For another 180 min, 250 mL of trisodium phosphate solution was added to adjust the pH to 6.8, to simulate the intestinal fluid. Stainless steel, spring-like sinkers were used to prevent tablets floating. The paddle speed was set at 50 rpm and the tests were conducted at 37 °C. Samples were withdrawn at predetermined time points and assayed spectrophotometrically at λ = 260 nm using a Jasco V-530 spectrophotometer (Tokyo, Japan) equipped with a 1 mm flow-through cuvette. The tests were carried out in triplicate and results represents averages with their standard deviations (SD).

## 4. Conclusions

In this article, we have analyzed the possibility of preparing 3D printed tablets characterized by different dose and dissolution profiles of ketoprofen using a single feedstock filament. The use of poly(vinyl alcohol) as a matrix-forming polymer allowed us to produce good-quality filaments. We started from the filaments containing 40% of ketoprofen and 4% of the disintegrant. The best disintegration functionality among the tested disintegrants was exhibited by crospovidone. Thus, we prepared filaments containing 4% of crospovidone, with ketoprofen loading up to 50%. In the case of 50% ketoprofen-loaded filaments, the plasticizing impact of the API led to an excessive increase in filament elasticity, which caused printing problems. For this reason, all further tests were carried out for the filament containing 40% of ketoprofen with 4% addition of crospovidone, which was characterized by a high content of API and good printability, which resulted in high reproducibility of the printlets. Obtained results prove the great potential of the application of the FDM printing method in the production of personalized medicines without the need to produce many filaments with different compositions.

We have proven that all the studied factors, i.e., the dose of the API, the spatial arrangement of the tablet infill, and the composition of the filament, significantly affect the release of ketoprofen from tested formulations. Although ease of dose modification is one of the most frequently mentioned advantages of applying printing technologies to produce tailored medicines, it can affect the drug dissolution rate. On the other hand, composition and structure optimization allowed us to produce tablets with the desired dosages and release profiles. Additionally, the HME and FDM processes resulted in the amorphization of the ketoprofen. Moreover, the analyses conducted two weeks after the printing of tablets showed no signs of crystallization of ketoprofen, indicating physical stability of the tested formulations.

## Figures and Tables

**Table 1:** Hot-melt extrusion parameters and filament properties (SD is the standard deviation).

Sample Name	Formulation	Die Temperature (°C)	Torque ± SD (Nm)	API Content ± SD (%)	Diameter ± SD (mm)	Young Modulus ± SD (N/mm^2^)
**Drug-Loaded Filaments**						
**F1**	KET20_K/CL	155	3.65 ± 0.12	20.99 ± 0.16	1.78 ± 0.02	2141.43 ± 75.39
**F2**	KET40	130	2.72 ± 0.29	37.88 ± 1.12	2.01 ± 0.02	618.08 ± 40.84
**F3**	KET40_K/CL	130	2.70 ± 0.04	40.23 ± 0.89	1.72 ± 0.03	501.13 ± 46.22
**F4**	KET40_VSOL	145	2.32 ± 0.12	40.52 ± 0.86	1.78 ± 0.07	466.85 ± 118.36
**F5**	KET40_VSTR	145	2.16 ± 0.10	39.48 ± 0.89	1.83 ± 0.02	502.99 ± 66.09
**F6**	KET50_K/CL	130	1.65 ± 0.05	48.39 ± 0.84	1.55 ± 0.06	187.93 ± 14.08
**Drug-Free Filament**						
**PF**	KIR_M	175	2.52 ± 0.38	-	1.63 ± 0.05	1296.61 ± 61.35

**Table 2:** Parameters of 3D printed tablets. RSD is the relative standard deviation.

Sample Name	Filament Formulation	Dose (mg)	Infill Density (%)	Tablet Weight (mg) ± RSD (%)	Printing Temperature (°C)
**T1**	KET20_K/CL	75	35	383.46 ± 0.55	185
**T2**	KET40	75	35	182.04 ± 0.29	180
**T3**	KET40_K/CL	75	35	181.73 ± 1.42	175
**T4**		100	20	244.31 ± 2.33	175
**T5**		100	35	253.23 ± 0.99	175
**T6**		100	50	244.93 ± 2.12	175
**T7**		150	35	375.71 ± 1.01	175
**T8**		150	50	378.98 ± 1.00	175
**T9**	KET40_VSOL	75	35	191.33 ± 0.56	175
**T10**	KET40_VSTR	75	35	179.50 ± 0.84	175
**T11**	KET50_K/CL	75	35	157.22 ± 0.39	175
**T12**	KET40_K/CL 2L	150	35/65	374.77 ± 2.27	175
**T13**	KET40_K/CL 3L	150	35/65/35	384.77 ± 1.23	175
**T14**	KET40_K/CL 1 + 1	100	35	507.84 ± 2.61	190
**T15**	KET40_K/CL 3 + 1	100	35	338.55 ± 3.64	190

**Table 3:** The properties of tablets analyzed with microCT.

Name	Dose (mg)	Length (mm)	Width (mm)	Height (mm)	Mass (mg)	Volume (mm^3^)	Surface (mm^2^)	Fraction	Printing *Path* Dimensions W × H (mm)
**T4**	100	19.88	10.04	3.39	243.8	186	1724	0.207	0.38 × 0.20
**T5**		19.90	10.07	2.51	246.9	186	1854	0.343	0.40 × 0.19
**T6**		19.88	10.09	1.80	241.8	186	1626	0.541	0.42 × 0.16
**T12**	150	19.89	9.97	3.15	382.8	289	2661	0.437	-
**T13**		19.89	10.06	3.13	383.3	298	2607	0.454	-

**Table 4:** The comparison of tablets attributes measured and calculated with different methods.

Name	µCT		Blender		Photocentric Studio	
	Volume (mm^3^)	Surface (mm^2^)	Volume (mm^3^)	Surface (mm^2^)	Volume (mm^3^)	Surface (mm^2^)
**T4**	186	1724	197	2034	197	2014
**T5**	186	1854	217	2132	217	2090
**T6**	186	1626	199	1759	199	1746
**T12**	289	2661	339	2862	339	2716
**T13**	298	2670	338	2823	338	2673

**Table 5:** Composition of the filaments.

Name	Formulation	Ketoprofen	Parteck^®^ MXP	Kollidon^®^ CL	Vivasol^®^	Vivastar^®^	Kollicoat^®^ IR	Mannitol
**Drug-Loaded Filaments**								
**F1**	KET20_K/CL	20%	76%	4%				
**F2**	KET40	40%	60%					
**F3**	KET40_K/CL	40%	56%	4%				
**F4**	KET40_VSOL	40%	56%		4%			
**F5**	KET40_VSTR	40%	56%			4%		
**F6**	KET50_K/CL	50%	46%	4%				
**Drug-Free Filament**								
**PF**	KIR_M						90%	10%
